# 6-Chloro-3,4-di­hydro-9*H*-carbazol-1(2*H*)-one

**DOI:** 10.1107/S1600536808023441

**Published:** 2008-07-31

**Authors:** M. Sridharan, K. J. Rajendra Prasad, A. Thomas Gunaseelan, A. Thiruvalluvar, R. J. Butcher

**Affiliations:** a Department of Chemistry, Bharathiar University, Coimbatore 641 046, Tamilnadu, India; b PG Research Department of Physics, Rajah Serfoji Government College (Autonomous), Thanjavur 613 005, Tamilnadu, India; c Department of Chemistry, Howard University, 525 College Street NW, Washington, DC 20059, USA

## Abstract

The carbazole unit of the title mol­ecule, C_12_H_10_ClNO, is not planar. The dihedral angle between the benzene and pyrrole rings is 1.35 (10)°. The cyclo­hexene ring adopts an envelope conformation. In the crystal structure, inter­molecular N—H⋯O hydrogen bonds form centrosymmetric dimers.

## Related literature

For a related structure with a non-planar carbazole unit, see: Sridharan *et al.* (2008 ▶).
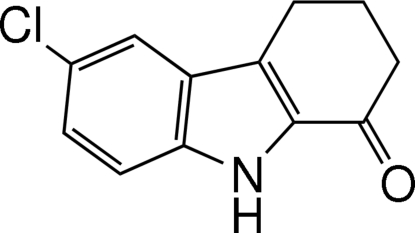


## Experimental

### 

#### Crystal data

C_12_H_10_ClNO
*M* *_r_* = 219.66
Monoclinic, 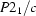

*a* = 10.4211 (5) Å
*b* = 5.6851 (3) Å
*c* = 17.0824 (10) Å
β = 100.239 (6)°
*V* = 995.93 (9) Å^3^
*Z* = 4
Mo *K*α radiation
μ = 0.35 mm^−1^
*T* = 200 (2) K
0.58 × 0.18 × 0.11 mm

#### Data collection

Oxford Diffraction R Gemini diffractometer
Absorption correction: multi-scan (*CrysAlis RED*; Oxford Diffraction, 2008 ▶) *T*_min_ = 0.923, *T*_max_ = 1.000 (expected range = 0.888–0.962)
10695 measured reflections
3909 independent reflections
1793 reflections with *I* > 2σ(*I*)
*R*_int_ = 0.072

#### Refinement

*R*[*F* ^2^ > 2σ(*F* ^2^)] = 0.062
*wR*(*F* ^2^) = 0.139
*S* = 0.88
3909 reflections
140 parameters
H atoms treated by a mixture of independent and constrained refinement
Δρ_max_ = 0.46 e Å^−3^
Δρ_min_ = −0.23 e Å^−3^

### 

Data collection: *CrysAlis CCD* (Oxford Diffraction, 2008 ▶); cell refinement: *CrysAlis RED* (Oxford Diffraction, 2008 ▶); data reduction: *CrysAlis RED*; program(s) used to solve structure: *SHELXS97* (Sheldrick, 2008 ▶); program(s) used to refine structure: *SHELXL97* (Sheldrick, 2008 ▶); molecular graphics: *ORTEP-3* (Farrugia, 1997 ▶); software used to prepare material for publication: *PLATON* (Spek, 2003 ▶).

## Supplementary Material

Crystal structure: contains datablocks global, I. DOI: 10.1107/S1600536808023441/sj2518sup1.cif

Structure factors: contains datablocks I. DOI: 10.1107/S1600536808023441/sj2518Isup2.hkl

Additional supplementary materials:  crystallographic information; 3D view; checkCIF report

## Figures and Tables

**Table 1:** Hydrogen-bond geometry (Å, °)

*D*—H⋯*A*	*D*—H	H⋯*A*	*D*⋯*A*	*D*—H⋯*A*
N9—H9⋯O1^i^	0.82 (2)	2.11 (2)	2.872 (2)	154 (2)

Symmetry code: (i) 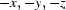.

**Fig. 1.**
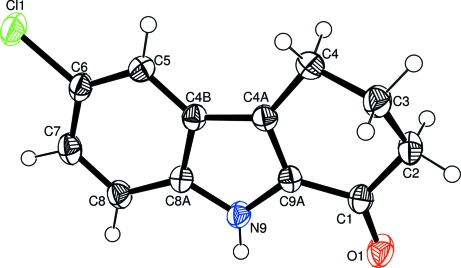
The molecular structure of the title compound, showing the atom-numbering scheme and displacement ellipsoids drawn at the 50% probability level. Hydrogen atoms are represented by spheres of arbitrary radius.

**Fig. 2.**
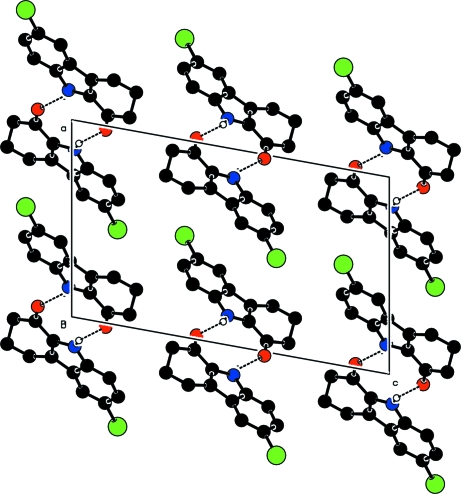
The molecular packing of the title compound, viewed down the b axis. Dashed lines indicate hydrogen bonds. H atoms not involved in hydrogen bonding have been omitted.

## supplementary crystallographic information

### Comment

Sridharan *et al.* (2008) have reported the crystal structure of 6-Methoxy-2,3,4,9-tetrahydro-1*H*-carbazol-1-one, in which the carbazole unit is not planar. The molecular structure of the title compound, with atomic numbering scheme, is shown in Fig. 1. The carbazole unit of the title molecule is not planar. The dihedral angle between the benzene ring and the pyrrole ring is 1.35 (10)°. The cyclohexene ring adopts an envelope conformation. Intermolecular N9—H9···O1 (-*x*, -*y*, -*z*) hydrogen bonds form centrosymmetric dimers in the crystal structure, Fig. 2.

### Experimental

A solution of 2-(2-(4-chlorophenyl)hydrazono)cyclohexanone (0.236 g, 0.001 mol) in a mixture of acetic acid (20 ml) and hydrochloric acid (5 ml) was refluxed on an oil bath pre-heated to 398–403 K for 2 h. The contents were then cooled and poured onto cold water with stirring. The brown solid which separated was purified by passing through a column of silica gel and eluting with (95:5 *v*/*v*) petroleum ether-ethyl acetate mixture to yield the title compound (0.153 g, 70%). This was recrystallized from ethanol.

### Refinement

The crystal used was very weakly diffracting particularly at high Bragg angles. The H atom bonded to N9 was located in a difference Fourier map and refined isotropically. Other H atoms were positioned geometrically and allowed to ride on their parent atoms, with C—H = 0.95–0.99 Å and *U*_iso_(H) = 1.2*U*_eq_(parent atom).

### Crystal data

**Table:**

C_12_H_10_ClNO	*F*_000_ = 456
*M**_r_* = 219.66	*D*_x_ = 1.465 Mg m^−^^3^
Monoclinic, *P*2_1_/*c*	Melting point: 475(1) K
Hall symbol: -P 2ybc	Mo *K*α radiation λ = 0.71073 Å
*a* = 10.4211 (5) Å	Cell parameters from 1948 reflections
*b* = 5.6851 (3) Å	θ = 4.6–34.7º
*c* = 17.0824 (10) Å	µ = 0.35 mm^−^^1^
β = 100.239 (6)º	*T* = 200 (2) K
*V* = 995.93 (9) Å^3^	Needle, colourless
*Z* = 4	0.58 × 0.18 × 0.11 mm

### Data collection

**Table:**

Oxford Diffraction R Gemini diffractometer	3909 independent reflections
Radiation source: fine-focus sealed tube	1793 reflections with *I* > 2σ(*I*)
Monochromator: graphite	*R*_int_ = 0.072
Detector resolution: 10.5081 pixels mm^-1^	θ_max_ = 34.7º
*T* = 200(2) K	θ_min_ = 4.6º
φ and ω scans	*h* = −16→15
Absorption correction: multi-scan(CrysAlis RED; Oxford Diffraction, 2008)	*k* = −9→9
*T*_min_ = 0.923, *T*_max_ = 1.000	*l* = −27→19
10695 measured reflections	

### Refinement

**Table:**

Refinement on *F*^2^	Secondary atom site location: difference Fourier map
Least-squares matrix: full	Hydrogen site location: inferred from neighbouring sites
*R*[*F*^2^ > 2σ(*F*^2^)] = 0.062	H atoms treated by a mixture of independent and constrained refinement
*wR*(*F*^2^) = 0.139	*w* = 1/[σ^2^(*F*_o_^2^) + (0.0611*P*)^2^] where *P* = (*F*_o_^2^ + 2*F*_c_^2^)/3
*S* = 0.88	(Δ/σ)_max_ = 0.001
3909 reflections	Δρ_max_ = 0.46 e Å^−^^3^
140 parameters	Δρ_min_ = −0.23 e Å^−^^3^
Primary atom site location: structure-invariant direct methods	Extinction correction: none

### Special details

**Table:**

Geometry. Bond distances, angles *etc*. have been calculated using the rounded fractional coordinates. All su's are estimated from the variances of the (full) variance-covariance matrix. The cell e.s.d.'s are taken into account in the estimation of distances, angles and torsion angles
Refinement. Refinement of *F*^2^ against ALL reflections. The weighted *R*-factor *wR* and goodness of fit *S* are based on *F*^2^, conventional *R*-factors *R* are based on *F*, with *F* set to zero for negative *F*^2^. The threshold expression of *F*^2^ > σ(*F*^2^) is used only for calculating *R*-factors(gt) *etc*. and is not relevant to the choice of reflections for refinement. *R*-factors based on *F*^2^ are statistically about twice as large as those based on *F*, and *R*- factors based on ALL data will be even larger.

### Fractional atomic coordinates and isotropic or equivalent isotropic displacement parameters (Å^2^)

**Table:**

	*x*	*y*	*z*	*U*_iso_*/*U*_eq_	
Cl1	0.51516 (5)	0.81197 (10)	−0.14449 (3)	0.0418 (2)	
O1	−0.02083 (14)	0.1402 (3)	0.10672 (9)	0.0403 (5)	
N9	0.14769 (15)	0.2338 (3)	−0.00949 (10)	0.0293 (5)	
C1	0.05496 (17)	0.3080 (3)	0.11410 (11)	0.0276 (5)	
C2	0.06417 (19)	0.4778 (4)	0.18253 (12)	0.0340 (6)	
C3	0.1973 (2)	0.5983 (4)	0.20336 (12)	0.0361 (6)	
C4	0.2388 (2)	0.7173 (4)	0.13273 (12)	0.0358 (7)	
C4A	0.22083 (17)	0.5536 (3)	0.06315 (11)	0.0258 (5)	
C4B	0.28050 (17)	0.5475 (3)	−0.00559 (11)	0.0251 (5)	
C5	0.36953 (17)	0.6955 (3)	−0.03472 (12)	0.0286 (5)	
C6	0.40706 (18)	0.6321 (3)	−0.10471 (12)	0.0292 (6)	
C7	0.36271 (19)	0.4274 (4)	−0.14660 (12)	0.0337 (6)	
C8	0.27603 (19)	0.2811 (4)	−0.11885 (12)	0.0318 (6)	
C8A	0.23419 (17)	0.3435 (3)	−0.04868 (11)	0.0260 (5)	
C9A	0.13871 (17)	0.3614 (3)	0.05784 (11)	0.0253 (5)	
H2A	−0.00399	0.59971	0.16903	0.0408*	
H2B	0.04640	0.39204	0.22991	0.0408*	
H3A	0.26364	0.47952	0.22511	0.0433*	
H3B	0.19391	0.71695	0.24535	0.0433*	
H4A	0.33155	0.76429	0.14653	0.0430*	
H4B	0.18603	0.86099	0.11876	0.0430*	
H5	0.40231	0.83389	−0.00698	0.0343*	
H7	0.39275	0.38971	−0.19443	0.0404*	
H8	0.24555	0.14153	−0.14666	0.0381*	
H9	0.1137 (19)	0.107 (4)	−0.0230 (12)	0.023 (5)*	

### Atomic displacement parameters (Å^2^)

**Table:**

	*U*^11^	*U*^22^	*U*^33^	*U*^12^	*U*^13^	*U*^23^
Cl1	0.0407 (3)	0.0437 (3)	0.0464 (3)	−0.0017 (2)	0.0224 (2)	0.0095 (3)
O1	0.0350 (7)	0.0496 (9)	0.0389 (8)	−0.0151 (7)	0.0138 (7)	−0.0069 (8)
N9	0.0308 (8)	0.0319 (9)	0.0269 (8)	−0.0096 (7)	0.0095 (7)	−0.0062 (8)
C1	0.0222 (8)	0.0333 (10)	0.0273 (9)	0.0006 (8)	0.0046 (7)	0.0012 (9)
C2	0.0336 (10)	0.0437 (12)	0.0271 (10)	−0.0017 (9)	0.0119 (9)	−0.0023 (10)
C3	0.0424 (11)	0.0397 (12)	0.0279 (10)	−0.0077 (9)	0.0109 (9)	−0.0051 (10)
C4	0.0459 (12)	0.0306 (11)	0.0333 (11)	−0.0057 (9)	0.0138 (10)	−0.0061 (10)
C4A	0.0265 (9)	0.0262 (9)	0.0250 (9)	0.0022 (7)	0.0051 (8)	0.0012 (9)
C4B	0.0255 (8)	0.0275 (10)	0.0223 (9)	0.0020 (7)	0.0043 (7)	0.0035 (8)
C5	0.0302 (9)	0.0269 (10)	0.0294 (9)	−0.0009 (8)	0.0071 (8)	0.0023 (9)
C6	0.0273 (9)	0.0319 (11)	0.0305 (10)	0.0040 (7)	0.0113 (8)	0.0085 (9)
C7	0.0355 (10)	0.0412 (12)	0.0266 (9)	0.0051 (9)	0.0117 (9)	0.0005 (10)
C8	0.0344 (10)	0.0348 (11)	0.0276 (10)	−0.0007 (8)	0.0097 (8)	−0.0046 (9)
C8A	0.0244 (8)	0.0302 (10)	0.0240 (9)	0.0004 (7)	0.0057 (7)	0.0006 (9)
C9A	0.0240 (8)	0.0287 (10)	0.0238 (9)	0.0026 (7)	0.0056 (7)	0.0007 (8)

### Geometric parameters (Å, °)

**Table:**

Cl1—C6	1.7460 (19)	C5—C6	1.371 (3)
O1—C1	1.231 (2)	C6—C7	1.401 (3)
N9—C8A	1.365 (2)	C7—C8	1.373 (3)
N9—C9A	1.377 (2)	C8—C8A	1.392 (3)
N9—H9	0.82 (2)	C2—H2A	0.9900
C1—C9A	1.441 (3)	C2—H2B	0.9900
C1—C2	1.506 (3)	C3—H3A	0.9900
C2—C3	1.531 (3)	C3—H3B	0.9900
C3—C4	1.512 (3)	C4—H4A	0.9900
C4—C4A	1.495 (3)	C4—H4B	0.9900
C4A—C4B	1.424 (3)	C5—H5	0.9500
C4A—C9A	1.381 (2)	C7—H7	0.9500
C4B—C8A	1.412 (2)	C8—H8	0.9500
C4B—C5	1.407 (3)		
			
Cl1···C4A^i^	3.5299 (19)	C8A···H2A^vi^	2.8900
Cl1···H7^ii^	3.1000	C9A···H3A	3.0000
Cl1···H4A^iii^	2.8900	C9A···H4B^v^	3.0400
O1···N9	2.924 (2)	H2A···H2B^x^	2.4900
O1···N9^iv^	2.872 (2)	H2A···C8^vi^	2.8900
O1···H4B^v^	2.6600	H2A···C8A^vi^	2.8900
O1···H9	2.83 (2)	H2B···H2A^xi^	2.4900
O1···H9^iv^	2.11 (2)	H3A···C9A	3.0000
N9···O1	2.924 (2)	H3A···C8^vii^	3.0300
N9···O1^iv^	2.872 (2)	H3A···H8^vii^	2.3400
C4A···Cl1^i^	3.5299 (19)	H3B···C7^xii^	3.0700
C5···C5^i^	3.549 (3)	H4A···Cl1^iii^	2.8900
C5···C6^i^	3.548 (3)	H4B···O1^xiii^	2.6600
C6···C5^i^	3.548 (3)	H4B···C1^xiii^	2.8800
C9A···C9A^vi^	3.566 (3)	H4B···C9A^xiii^	3.0400
C1···H4B^v^	2.8800	H7···Cl1^xiv^	3.1000
C3···H8^vii^	2.8700	H8···C3^ix^	2.8700
C7···H3B^viii^	3.0700	H8···H3A^ix^	2.3400
C8···H2A^vi^	2.8900	H9···O1	2.83 (2)
C8···H3A^ix^	3.0300	H9···O1^iv^	2.11 (2)
			
C8A—N9—C9A	108.53 (15)	C1—C9A—C4A	124.38 (17)
C9A—N9—H9	127.6 (14)	N9—C9A—C1	125.83 (16)
C8A—N9—H9	123.7 (14)	N9—C9A—C4A	109.78 (16)
O1—C1—C9A	123.29 (17)	C1—C2—H2A	109.00
O1—C1—C2	121.85 (17)	C1—C2—H2B	109.00
C2—C1—C9A	114.85 (16)	C3—C2—H2A	109.00
C1—C2—C3	113.41 (16)	C3—C2—H2B	109.00
C2—C3—C4	112.99 (17)	H2A—C2—H2B	108.00
C3—C4—C4A	110.04 (18)	C2—C3—H3A	109.00
C4B—C4A—C9A	106.44 (16)	C2—C3—H3B	109.00
C4—C4A—C4B	131.28 (17)	C4—C3—H3A	109.00
C4—C4A—C9A	122.24 (17)	C4—C3—H3B	109.00
C5—C4B—C8A	119.51 (17)	H3A—C3—H3B	108.00
C4A—C4B—C8A	106.93 (15)	C3—C4—H4A	110.00
C4A—C4B—C5	133.57 (17)	C3—C4—H4B	110.00
C4B—C5—C6	117.36 (16)	C4A—C4—H4A	110.00
Cl1—C6—C5	119.30 (14)	C4A—C4—H4B	110.00
Cl1—C6—C7	117.70 (15)	H4A—C4—H4B	108.00
C5—C6—C7	123.00 (17)	C4B—C5—H5	121.00
C6—C7—C8	120.30 (19)	C6—C5—H5	121.00
C7—C8—C8A	117.96 (19)	C6—C7—H7	120.00
N9—C8A—C8	129.85 (17)	C8—C7—H7	120.00
C4B—C8A—C8	121.86 (17)	C7—C8—H8	121.00
N9—C8A—C4B	108.29 (16)	C8A—C8—H8	121.00
			
C9A—N9—C8A—C4B	−0.6 (2)	C4—C4A—C4B—C8A	175.46 (19)
C9A—N9—C8A—C8	179.52 (19)	C9A—C4A—C4B—C5	178.3 (2)
C8A—N9—C9A—C1	178.12 (17)	C4B—C4A—C9A—N9	1.7 (2)
C8A—N9—C9A—C4A	−0.7 (2)	C4B—C4A—C9A—C1	−177.15 (17)
C2—C1—C9A—N9	−179.78 (17)	C4A—C4B—C8A—N9	1.6 (2)
C2—C1—C9A—C4A	−1.1 (3)	C5—C4B—C8A—C8	1.3 (3)
O1—C1—C2—C3	153.79 (19)	C4A—C4B—C8A—C8	−178.47 (18)
C9A—C1—C2—C3	−27.7 (2)	C5—C4B—C8A—N9	−178.63 (16)
O1—C1—C9A—N9	−1.3 (3)	C4A—C4B—C5—C6	179.7 (2)
O1—C1—C9A—C4A	177.38 (18)	C8A—C4B—C5—C6	0.0 (3)
C1—C2—C3—C4	53.7 (2)	C4B—C5—C6—Cl1	178.90 (14)
C2—C3—C4—C4A	−48.1 (2)	C4B—C5—C6—C7	−1.2 (3)
C3—C4—C4A—C4B	−157.05 (19)	Cl1—C6—C7—C8	−178.99 (16)
C3—C4—C4A—C9A	20.1 (3)	C5—C6—C7—C8	1.1 (3)
C4—C4A—C9A—N9	−176.05 (17)	C6—C7—C8—C8A	0.2 (3)
C4—C4A—C9A—C1	5.1 (3)	C7—C8—C8A—N9	178.52 (19)
C9A—C4A—C4B—C8A	−2.0 (2)	C7—C8—C8A—C4B	−1.4 (3)
C4—C4A—C4B—C5	−4.2 (4)		

Symmetry codes: (i) −*x*+1, −*y*+1, −*z*; (ii) −*x*+1, *y*+1/2, −*z*−1/2; (iii) −*x*+1, −*y*+2, −*z*; (iv) −*x*, −*y*, −*z*; (v) *x*, *y*−1, *z*; (vi) −*x*, −*y*+1, −*z*; (vii) *x*, −*y*+1/2, *z*+1/2; (viii) *x*, −*y*+3/2, *z*−1/2; (ix) *x*, −*y*+1/2, *z*−1/2; (x) −*x*, *y*+1/2, −*z*+1/2; (xi) −*x*, *y*−1/2, −*z*+1/2; (xii) *x*, −*y*+3/2, *z*+1/2; (xiii) *x*, *y*+1, *z*; (xiv) −*x*+1, *y*−1/2, −*z*−1/2.

### Hydrogen-bond geometry (Å, °)

**Table:**

*D*—H···*A*	*D*—H	H···*A*	*D*···*A*	*D*—H···*A*
N9—H9···O1^iv^	0.82 (2)	2.11 (2)	2.872 (2)	154 (2)

Symmetry codes: (iv) −*x*, −*y*, −*z*.
